# Frequency of vasovagal reactions in whole blood donation and contributing factors

**DOI:** 10.3892/mi.2025.221

**Published:** 2025-02-18

**Authors:** Sinan Demircioğlu, Ali Türk, Atilla Tomruk, Havva Demircioğlu, Atakan Tekinalp

**Affiliations:** 1 Department of Hematology, Faculty of Medicine, Necmettin Erbakan University, Konya 42090, Turkey; 2 Transfusion Center, Faculty of Medicine, Necmettin Erbakan University, Konya 42090, Turkey; 3 Meram Family Health Center No. 25, Konya 42090, Turkey

**Keywords:** whole blood, blood donation, vasovagal reactions, transfusion, hemovigilance

## Abstract

The aim of the present study was to identify the frequency of vasovagal reactions (VVRs) and the factors influencing their occurrence. For this purpose, a total of 742 donors who applied for whole blood donation at a blood center were included in the study. The frequency of VVR and its association with donor-related factors were assessed. The results revealed that the median age of the donors was 36 years, with 9.8% female and 90.2% male donors. VVRs were observed in 4.9% of the donors. The median age of the donors who experienced VVRs was found to be lower than that of those who did not experience VVRs. The incidence of VVRs was higher in first-time donors. The median blood donation rate for donors who experienced VVRs was 2, compared to 4 for those who did not, and the difference was statistically significant. On the whole, the present study demonstrates that the frequency of VVRs is higher in first-time blood donors, and that the blood donation rate is lower for those who experience VVRs. Preventive measures for VVRs, particularly for first-time donors, could increase the rate of blood donation.

## Introduction

Although blood donation is generally a safe procedure, undesirable reactions can occur during or after the collection of whole blood or blood components. The most common adverse event is a vasovagal reaction (VVR; or vasovagal syncope). VVRs are considered to be triggered by various physical (e.g., standing up after losing 500 ml blood) and psychological stimuli (e.g., pain, stress and fear) (1). During a VVR, there is a decrease in the arterial blood pressure and cerebral perfusion of the donor, which reduces the blood flow to the brain (2). The highest frequency of VVRs in whole blood donors occurs during needle removal and when leaving the donation chair (3). A small proportion (9-12%) of reported VVRs occurs after the donor leaves the center. However, the number of off-site reactions is often underestimated due to underreporting by donors (4). The symptoms of VVRs include general weakness, dizziness, pallor, sweating, anxiety and nausea; additionally, some donors may experience more severe symptoms, such as a transient loss of consciousness (syncope), convulsions, or incontinence (5). Fainting and falling can result in accidental injuries. VVRs also prevent individuals from donating again, decreasing the likelihood of repeat donations by >50% (6,7).

VVRs affect donor safety and future donation behavior. They also negatively affect donor waiting times, appointment management and the number of completed blood collections (7). Therefore, risk factors for VVR need to be identified, and preventive policies need to be developed. The present study aimed to investigate the frequency and causes of VVR in whole blood donors at the Transfusion Center, Faculty of Medicine, Necmettin Erbakan University.

## Subjects and methods

### Donor information

A total of 742 donors who applied for whole blood donation at the Necmettin Erbakan University Transfusion Center (Konya, Turkey) were included in the study. The present study was conducted between September and October, 2024. Donor eligibility was evaluated according to the National Blood and Blood Component Preparation, Use, and Quality Assurance Guide (8). All donors were provided with information about the donation process prior to the donation. Potential complications were explained. At this stage, those who decided to withdraw from blood donation were not pressured to continue. Following the donation, donors were taken to the rest area where they were served fruit juice and encouraged to sit and rest for 15 min. In cases of VVR, necessary interventions were performed by the doctor. To assess the health status of the donors and determine their eligibility for blood donation, blood pressure, body temperature, and pulse rate were measured prior to the donation.

An average of 450±45 cc of whole blood was collected from the donors. Donor age, sex, comorbidities, fasting status, time since last meal, blood donation history, leukocyte count, hemoglobin level and platelet count were recorded. The frequency and timing of VVRs were determined, and the association between VVRs and the donor characteristics was analyzed. The present study was approved by the Necmettin Erbakan University, Ethics Committee for Non-Pharmaceutical and Non-Medical Device Research (2024-5381). Written consent was obtained from the participants.

### Statistical analysis

Statistical analysis was performed using the SPSS IBM program, version 23.0 (IBM Corp.). The distribution characteristics of continuous variables were evaluated using the Kolmogorov-Smirnov test. Descriptive statistics for normally distributed data are expressed as the mean ± standard deviation, while non-normally distributed data are presented as the median (range). Group comparisons for continuous variables were conducted using the Mann-Whitney U test. Categorical variables are expressed as percentages (%) and compared using the Fisher's test. Values of P<0.05 were considered to indicate a statistically significant difference.

## Results

A total of 742 blood donors were evaluated. The median age of the donors was 36 years (range, 18-67 years), with 69 (9.8%) female and 673 (90.2%) male donors. The median age of the donors was similar between the male and female donors (35 years vs. 36 years).

**Figure 1:**
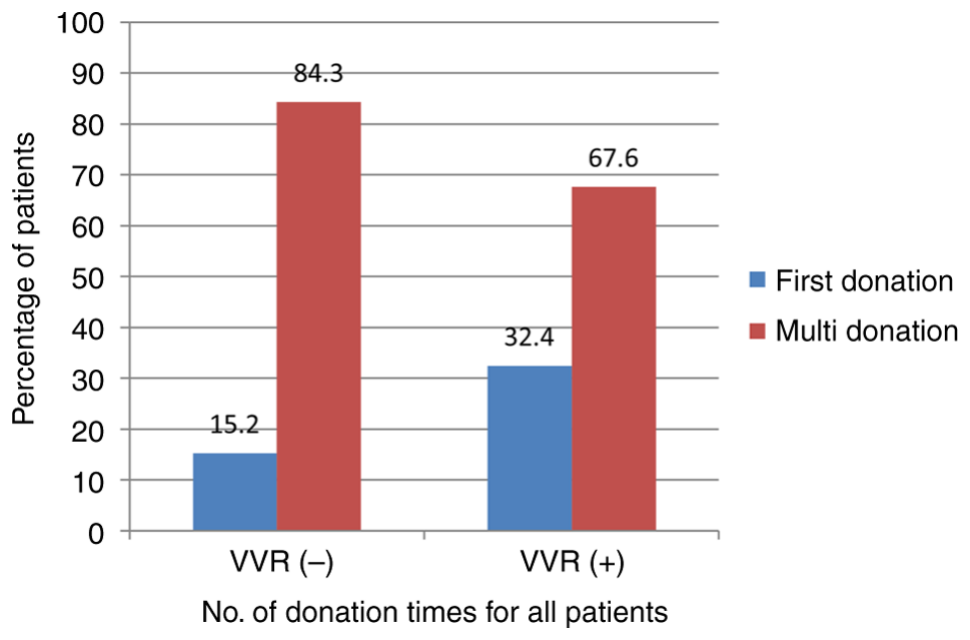
The frequency of VVR development based on donation history. VVR, vasovagal reaction.

**Figure 2:**
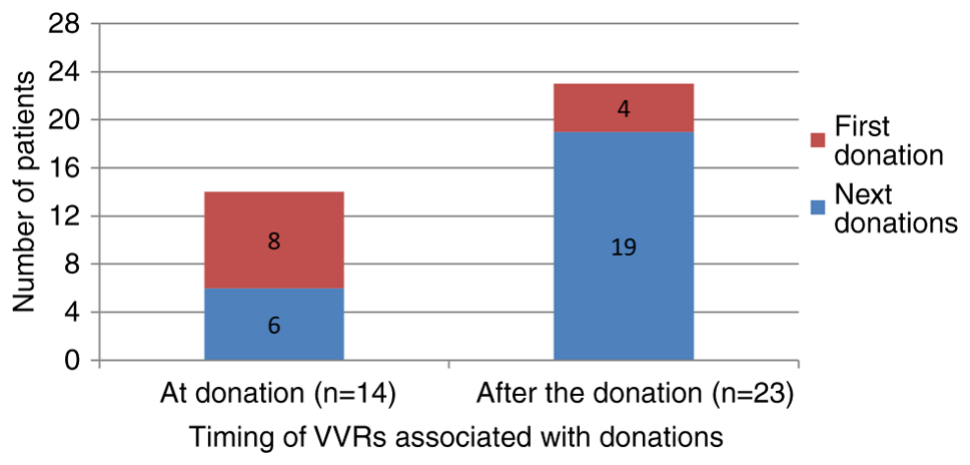
Association between the timing of VVR development and donation history. VVR, vasovagal reaction.

VVR was detected in 37 (4.9%) donors. A total of 7 out of 62 females (10.1%) and 30 out of 643 males (4.45%) developed VVR, with no significant difference. The median age of the donors who experienced VVR was significantly lower than that of those who did not (P=0.04). Of note, 16% of the donors (119 individuals) were first-time donors. VVRs developed in 10% (12 individuals) of the first-time donors. First-time blood donors had a higher incidence of VVR (32.4% vs. 15.2%, P=0.010) (Fig. 1). The median blood donation rate for donors who experienced VVRs was 2, compared to 4 for those who did not, and this difference was statistically significant (P<0.001) (Table I). No significant effects of fasting status, sex, comorbidities, leukocyte count, hemoglobin, hematocrit, or platelet count on VVRs were found (Table I). As regards the timing of VVRs, among the 37 donors who experienced VVRs, 14 (37.8%) had it during the donation, and 23 (62.2%) after the donation. First-time donors had a higher rate of VVR during the donation (57.1% vs. 17.4%, P=0.027) (Table I and Fig. 2).

## Discussion

In blood donation, moderate VVRs occur in 1.4 to 7% of individuals, and severe VVRs occur in 0.1 to 0.5% of cases (4,9-18). The present study found a VVR rate of 4.9%, which is similar to that observed in the literature. The hospitalization rate due to blood donation complications has been reported as 1/198,000, with two-thirds of these events associated with VVRs. Falls leading to injuries have been found to be the most significant cause in female donors (4). None of the donors in the present study required hospitalization.

The following risk factors have been shown to be associated with an increased risk of VVRs: The female sex (7,19,20), a low body weight (21,22), a low estimated blood volume (9,20,23), a younger age (7,9,19,23), first-time donation (7,19,22,23), a low resting blood pressure (21), insufficient sleep prior to donation (24,25), donation site (23) and a history of symptoms during previous donations (26,27). The frequency of VVRs has been found to be higher in first-time, young and female donors (21,28,29). Additionally, donors who experienced VVRs had a significantly lower total blood donation count. This may be due to regular donations from those who did not experience VVRs in previous donations, while those who experienced VVRs tend to avoid further donations. Unlike other studies, the female sex was not found to be a risk factor for VVRs in the present study, possibly due to the smaller number of female donors. The primary reason for the low number of female donors is iron deficiency anemia, which develops in menstruating women. Iron deficiency affects 29 to 58% of healthy females of reproductive age, while the prevalence of iron deficiency anemia ranges from 27 to 47% (30-32). Another reason is the reluctance to select women who have had pregnancies as donors in Turkey. The female donor rate and the frequency of VVR occurrence among female donors in different countries is presented in Table II. The frequency of female donors varies greatly across countries. Upon reviewing these studies, it appears that factors, such as the level of development of the countries and religious beliefs play a prominent role in influencing the rate of female donors. Similar to these countries, in the authors' country, Turkey, the socio-cultural situation may be another main reason for women donating less in certain segments of society.

Setting weight limits for blood donation is critical for protecting donors from adverse effects, particularly vasovagal episodes and anemia. It has been shown that a low body weight and low blood volume are independent predictors of VVRs (10,21). Trouern-Trend *et al* (21) demonstrated that a low body weight increases the risk of developing VVRs. They demonstrated that VVRs developed in 4.6% of individuals with a body weight <120 lb and in 0.4% of those with a body weight >210 lb (21). Another study found that body weight was a significant determinant of the rate of vasovagal reactions in first-time blood donors (22). In general, it is accepted that the total volume of donated blood should not exceed 13% of the blood volume of the donor. For example, a donor must weigh at least 45 kg to donate 350 ml (±10%) of blood, and at least 50 kg to donate 450 ml (±10%) of blood (38). There is no specific upper weight limit for blood donation. In Turkey, the donor selection criteria require that the donor weighs >50 kg (8).

Psychological stresses, such as pre-donation sleep duration, a history of fainting epidodes, anxiety traits, a fear of blood and injury, and a fear of needles have been associated with VVRs (24,34-37). In their study, Takanashi *et al* (24) compared the records of 4,924 Japanese donors who experienced VVR with a control group of 43,948 donors who did not experience any such complications which were related to donation. As observed in their study, for both male and female donors, factors such as being a first-time donor, having a pre-donation pulse rate of ≥90 beats per minute, a diastolic blood pressure ≤70 mmHg, <6 h of sleep (compared to >8 h of sleep), not eating within the previous 4 h, and factors such as age, sex, body mass index, pulse rate and systolic blood pressure were all significantly associated with an increased risk of having VVRs (24).

The underlying mechanisms of VVRs include the orthostatic effects of hypovolemic static status (i.e., a decrease in blood pressure when standing up following the donation) and psychological stress related to the procedure (e.g., pain associated with needle insertion or phlebotomy) (38). Due to the complexity of the mechanisms involved, current prevention strategies target both the physiological and psychological aspects of the reaction (39).

Various strategies have been proposed to prevent VVRs. For example, the World Health Organization (WHO) recommends pre-donation hydration and the application of active muscle tension (AMT) to increase blood flow and blood pressure. The EU Domaine Project supports pre-donation hydration and AMT, as well as caffeine loading, distraction techniques, supportive care, and educational materials (40-42). Blood services worldwide have adopted various strategies to prevent VVRs, with water loading and AMT being the most common (43,44).

Compared to the empirical literature focusing on pre-donation hydration and AMT, only a limited number of studies have addressed the psychological aspects of the donation and their association with vasovagal symptoms. Studies evaluating psychological methods primarily focus on reducing stress or anxiety by providing distraction or social support during the procedure (39). In a previous study, the effectiveness of audiovisual distraction for first-time donors was investigated by classifying individuals based on their coping styles as monitoring (attending to the situation) or blunting (distracting, denying, or reinterpreting the situation) (45). Donors with blunting coping styles who were provided with a distracting environment reported significantly lower self-reported vasovagal symptoms compared to participants in the untreated control condition (45). Hanson and France (46) demonstrated that donors who were accompanied by a trained research assistant for support had lower VVR levels and were more willing to donate again compared to donors who did not receive support.

**Figure 3:**
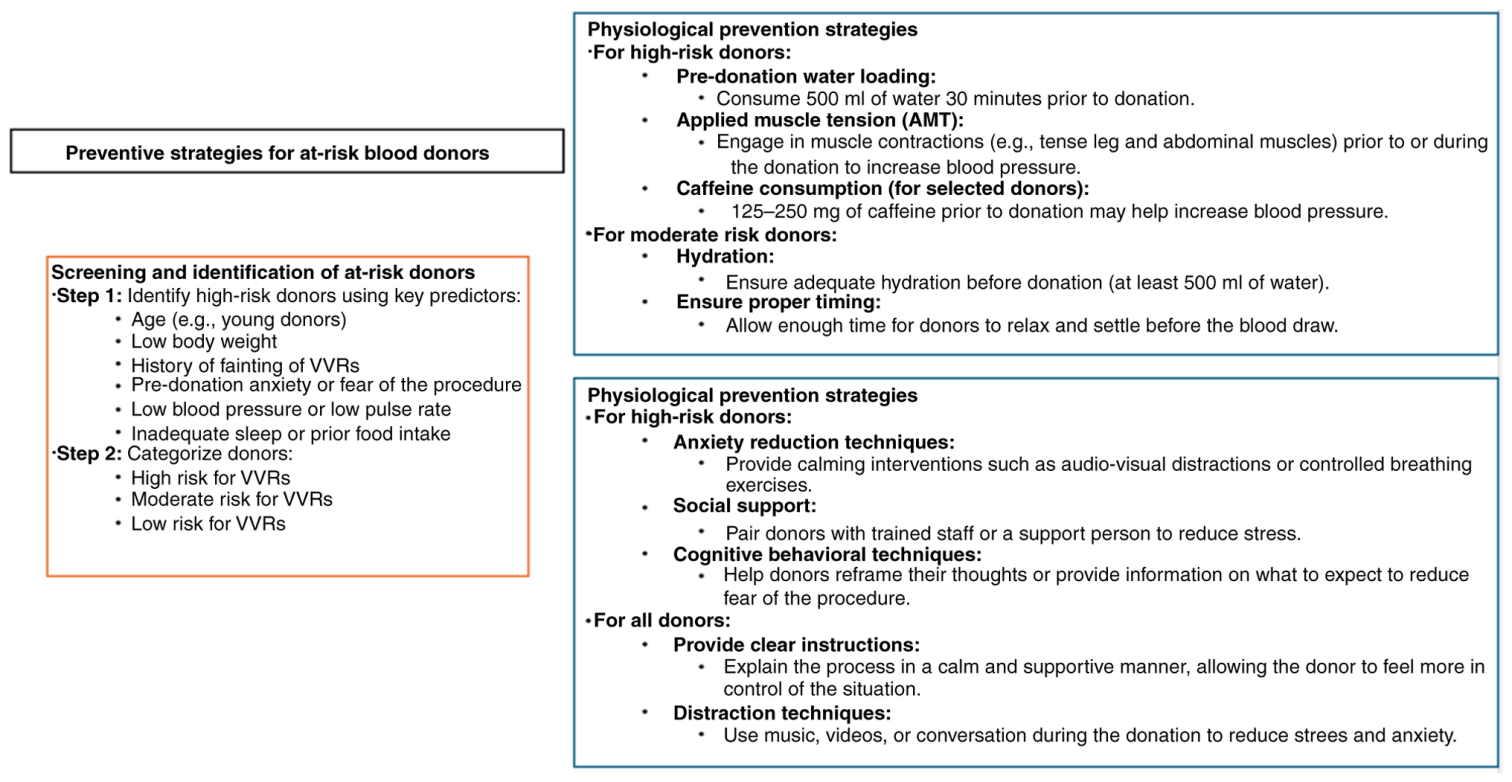
Preventive strategies for at-risk blood donors.

VVRs can be minimized with simple precautions. First and foremost, donor education is essential. Donors should be advised to avoid coming on an empty stomach, refrain from smoking before and after donation, avoid standing up immediately after donation (and instead sit up first), and ensure adequate fluid intake before and/or after donation. These measures will prevent the majority of cases of VVRs. However, despite these precautions, VVRs may still occur, particularly in first-time blood donors, due to psychological stress. Donors who exhibit signs of psychological stress should receive psychological support before proceeding with blood donation. Therefore, blood donation centers should have trained personnel capable of providing psychological support. Another potential factor in reducing VVR is ensuring that the blood donation environment is spacious, clean, well-ventilated, and comfortable. Additionally, a friendly attitude from the staff, effective communication, reassuring the donor, and good technical skills are important factors in reducing VVR (Fig. 3).

A limitation of the present study is the lack of the evaluation of body weight, blood pressure, body temperature, oxygen saturation and psychological stress levels prior to donation. These factors could provide valuable insight into the overall health and well-being of donors, potentially affecting the donation process and outcomes. In future studies on this topic, the inclusion of these parameters would enhance the value of the research and offer a more comprehensive understanding of the factors influencing donation. Another limitation of the present study is the significantly lower number of females compared to males. However, this is not a choice of the authors, but rather a result of the general tendency of females in Turkey to donate blood.

As a result, in the present study, the frequency of VVRs was found to be 4.9%, and it was observed that VVRs developed more frequently in younger individuals and first-time donors. Although numerous intervention studies have evaluated the effectiveness of physiological and psychological methods in preventing VVRs and/or vasovagal symptoms, questions regarding the efficacy of these techniques remain. A reasonable approach may be to apply both physiological and psychological preventive methods to young first-time donors to prevent them from experiencing VVRs. This is due to the fact that those who experience VVRs during their first blood donation tend to avoid donating again, which leads to a decrease in the number of blood donors.

## Figures and Tables

**Table I:** Epidemiological and laboratory comparison of the donors with and without VVRs.

Parameter	VVR (-) (n:705)	VVR (+) (n:37)	P-value
Sex, n (%)			0.072^a^
Female	62 (8.8)	7 (18.9)	
Male	643 (91.2)	30 (81.1)	
Additional disease, n (%)			NS^a^
Yes	18 (2.6)	0	
No	687 (97.4)	37(100)	
Fasting status, n (%)			NS^a^
Yes	11 (1,6)	0	
No	694 (98.4)	37	
Donation history			**0.010^a^**
First-time	107 (15.2)	12 (32.4)	
Repeat	598 (84.8)	25 (67.6)	
Age (median, range)	36 (18-67)	30 (18-56)	**0.04^b^**
Time since last meal (h)	2 (0.5-12)	1 (0.5-4)	1.29^b^
No. of blood donations	4 (0-80)	2 (0-20)	**<0.001^b^**
Leukocyte count (mm^3^)	7,745 (4,100-11,800)	7,550 (5,400-12,500)	0.611^b^
Hemoglobin (g/dl)	15.3 (12,4-18)	15 (12.5-17.5)	0.072^b^
Hematocrit (%)	44.6 (36.1-52.1)	43.6 (28.2-50.9)	0.064^b^
Platelet count (x10^3^/µl)	277.5 (112-475)	294 (122-393)	0.79^b^
No. of donation times, n (%)			0.010^a^
First donation	107 (15.2)	12 (32.4)	
Multiple donations	598 (84.3)	25 (67.6)	
The timing of VVRsc, n (%)			0.027^a^
During the donation (n=14)			
First donation			
Multiple donations		8 (57.1)	
After the donation (n=23)		6 (42.9)	
First donation			
Multiple donations		4 (17.4)	
		19 (82.6)	

P-values in bold font indicate statistically significant differences (P<0.05). NS, not significant. Data were analyzed using

^a^Fisher's exact test or

^b^Mann-Whitney U test. VVR, vasovagal reaction.

^c^Regarding patients with VVRs. VVRs, vasovagal reactions.

**Table II:** Incidence of vasovagal reactions in blood donation in different countries worldwide.

Country (year of publication)	Sample size	% of bood donation in females	% of females developing VVR	% of blood donation in first donors	% of VVR improving in first-time donors	Incidence of VVR (%)	(Refs.)
USA (2008)	422,231	57.9	1.86	24.6	2.75	1.43	(9)
USA (2010)	793,293	48	0.65	21	0.89	0.41	(4)
Saudi Arabia (2017)	18,936	1.4	1.2	47	1.6	1.1	(12)
Italy (2009)	183,855	8.6		19.5		0.19	(13)
India (2014)	88,201	3.9		35.8		1.23	(14)
Pakistan (2016)	41,579	0.2	0			1.03	(15)
Germany (2015)	928,411	47.8		8.2	2.78	0.76	(16)
Iran (2011)	5,285	4.4	8.6	18	5.6	2	(17)
Brazil (2012)	724,861	30	3.39	31.5	4.28	2.2	(18)
Turkey (2025)	742	9.8	10.1	16	10	4.9	Present study

## Data Availability

The data generated in the present study may be requested from the corresponding author.
